# A Multiband Millimeter-Wave Rectangular Dielectric Resonator Antenna with Omnidirectional Radiation Using a Planar Feed

**DOI:** 10.3390/mi14091774

**Published:** 2023-09-16

**Authors:** Tarek S. Abdou, Salam K. Khamas

**Affiliations:** Communications Research Group, Department of Electronic and Electrical Engineering, University of Sheffield, Sheffield S1 3JD, UK; tsabdou1@sheffield.ac.uk

**Keywords:** omnidirectional antenna, dielectric resonator antenna, multiband, mmWave communications

## Abstract

In this study, a millimeter-wave (mmWave) dielectric resonator antenna (DRA) with an omnidirectional pattern is presented for the first time. A key feature of the proposed design is the utilization of a planar feed network to achieve omnidirectional radiation from a rectangular DRA, which has not been reported previously in the open literature. In addition, the proposed antenna offers multiband operation with different types of radiation patterns. The degenerate TE_121_/TE_211_ modes were excited at 28.5 GHz with an overall internal electromagnetic field distribution that was similar to that of the HEM_21δ_ mode of a cylindrical DRA. The achieved omnidirectional bandwidth and gain were 1.9% and 4.3 dBi, respectively. Moreover, broadside radiation was achieved by exciting the TE_111_ fundamental mode at 17.5 GHz together with the resonance of the feeding ring-slot at 23 GHz. The triple-band operation offers a highly versatile antenna that can be utilized in on-body and off-body communications. Furthermore, the proposed design was validated through measurements, demonstrating good agreement with simulations.

## 1. Introduction

Over recent years, the evolution of mmWave communication systems has led to more rigorous requirements for antenna designs such as high gain, wide and multiband operation, as well as pattern diversity. DRAs have the potential of addressing these requirements due to well-known advantages such as high radiation efficiency, wide impedance bandwidth, and design flexibility. Therefore, mmWave DRAs have received increased research interest with a focus on broadside radiation [1,2,3,4]. On the other hand, omnidirectional radiation is desired for 5G and Beyond 5G (B5G) communication systems to increase the coverage area in various applications such as on-body communications as well as device-to-device short-distance communications [5]. Therefore, several studies have been reported on the design of mmWave omnidirectional antennas [6,7]. However, an omnidirectional mmWave DRA has not been reported previously, which is in sharp contrast with the numerous published studies on the design of omnidirectional DRAs at lower frequencies with a focus on exciting specific transverse magnetic (TM) and transverse electric (TE) modes to achieve the required pattern.

For example, an omnidirectional cylindrical DRA was proposed by exciting the ${TE}_{01\delta }$ and ${TM}_{01\delta }$ resonance modes at 3.87 GHz and 4.02 GHz, respectively, using a central coaxial probe feed in [8]. Moreover, a multiband, multisense, circularly polarized hybrid patch/DRA omnidirectional antenna was reported by exciting the ${TM}_{02}$ and ${TM}_{011}$ resonance modes for the patch at 2 GHz and DRA at 2.6 GHz, respectively [9]. In a more recent study, a wideband filtering omnidirectional cylindrical DRA was presented using a hybrid feed that consisted of a coaxial probe and metallic disk to excite the ${TM}_{01\delta }$ and ${TM}_{013}$ DRA resonance modes at 2.19 GHz and 3.37 GHz, respectively, in [10]. Further, a coaxial probe-fed omnidirectional hemispherical DRA was proposed by exciting the ${TM}_{101}$ resonance mode at 3.7 GHz in [11]. Another probe-fed omnidirectional hemispherical DRA was designed for a wireless capsule endoscope system by exciting the ${TM}_{01\delta }$ and ${TE}_{01\delta }$ resonance modes for multipolarization at 2.45 GHz in [12]. Moreover, a probe-fed omnidirectional rectangular DRA with a square cross section was designed by exciting the quasi-TM_011_ mode at 2.4 GHz for linear and circular polarizations in [13]. Furthermore, innovative rectangular multifunction glass DRAs were reported with the capability of achieving linearly and circularly polarized omnidirectional radiation patterns by exciting the quasi-TM_011_ mode at 2.4 GHz in [14]. Similarly, the higher-order degenerate ${TE}_{121}^{x}$ and ${TE}_{211}^{y}$ modes were excited at 3.6 GHz, with equal amplitude and phase, to achieve omnidirectional radiation from a rectangular DRA in [15,16]. Subsequently, a multiband probe-fed omnidirectional rectangular DRA was proposed, where the ${TE}_{121}^{x}$ and ${TE}_{211}^{y}$ resonance modes were excited at 3.5 GHz together with the ${TE}_{141}^{x}$ and ${TE}_{411}^{y}$ resonance modes at 5.8 GHz, in [16].

It should be noted that in all the above-mentioned studies, omnidirectional radiation was attained using a centrally located coaxial feeding probe to excite the required resonance modes. On the other hand, owing to their capability of supporting various types of modes when placed above a ground plane, cylindrical DRAs have successfully been utilized recently with planar feed networks to achieve omnidirectional radiation patterns. For example, an omnidirectional cylindrical DRA with a planar feed of a shorted microstrip cross was demonstrated by exciting the ${TM}_{01\delta }$ and ${TM}_{011+\delta }$ resonance modes at ~2.4 GHz to achieve circular polarization diversity [17]. The first attempt to utilize a ring-slot aperture to feed an omnidirectional cylindrical DRA was proposed by exciting the ${TM}_{01\delta }$ fundamental resonance mode at 2.4 GHz [18]. Furthermore, a pattern diversity cylindrical DRA was proposed using a meander line-loaded annular slot to excite the TM_01δ_ mode in combination with a differential strip to excite the HEM_11δ_ for omnidirectional and broadside radiations, respectively, at 2.4 GHz [19]. Moreover, four linear stubs were utilized to excite the ${TM}_{01\delta }$ and ${TM}_{011+\delta }$ resonance modes of an omnidirectional cylindrical DRA to achieve circular polarization at 5.8 GHz [20]. In a more recent study, arched-aperture feeding was employed in the design of a wideband omnidirectional cylindrical DRA by merging the bandwidth of the excited TM_01δ_ and TM_02δ_ resonance modes at ~5.8 GHz [21].

A rectangular DRA with a square cross section supports quasi-TM modes [22] that have been traditionally excited using a coaxial feeding probe to achieve omnidirectional radiation [13,14,15,16]. It is well-known that a coaxial feeding probe requires a hole to be drilled inside the DRA, which is impractical at mmWave frequencies due to the physically small DRA size. Furthermore, the probe’s reactance can be large at millimeter-wave frequencies. Moreover, the power handling capacity of the probe is reduced at higher frequencies, leading to signal degradation and power dissipation [18,23]. Therefore, aperture–slot coupling is preferred to excite a DRA at higher operating frequencies as it provides a high level of isolation between the antenna and the planar feed network. On the other hand, compared to the cylindrical counterpart, a rectangular DRA offers an additional degree of design freedom together with simpler fabrication due to the planar sides. Therefore, an alternative noninvasive feeding approach needs to be utilized to design a mmWave omnidirectional rectangular DRA. Such a design is proposed in this study, where a ring-slot aperture is utilized to excite the required modes. In addition to the planar feed, the proposed antenna offers another advantage of multiband operation with two types of radiation patterns: broadside and omnidirectional. The first is achieved by exciting the fundamental TE_111_ mode at 17.5 GHz as well as a slot resonance at 23 GHz. An omnidirectional pattern is achieved by exciting the ${TE}_{121}^{x}$ and ${TE}_{211}^{y}$ higher-order degenerate modes. It should be noted that all the reported dual-band DRA designs radiate either broadside or omnidirectional patterns in both bands. As a result, the proposed DRA can be employed simultaneously for off-body and on-body applications, for example, by utilizing the broadside and omnidirectional patterns, respectively. A common problem with existing on-body antennas is the reduced radiation efficiency due to the impact of the human body, especially at mmWave frequencies. However, the utilization of on-body omnidirectional DRA can help in achieving more efficient on-body antennas, which necessitates the design of a planar feeding network.

This article is organized as follows: Section 2 presents the proposed DRA configuration. Section 3 investigates the excitable DRA modes at a frequency range of 20–30 GHz. Section 4 is focused on the design of the planar feed network. Section 5 presents an analysis of the performance of the on-body mmWave DRA. Section 6 presents the measured results that agree closely with the simulations and Section 7 is focused on the conclusions. All the simulations are implemented using CST microwave studio.

## 2. Antenna Configuration

**Figure 1:**
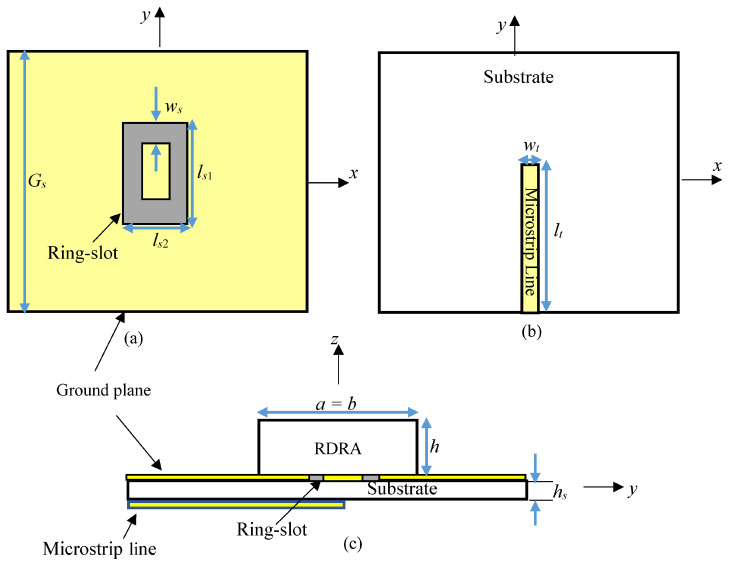
The proposed omnidirectional DRA: (**a**) ground plane with an etched ring-slot; (**b**) bottom view; (**c**) side view.

The DRA was designed using a square cross section to facilitate the excitation of the required degenerate modes for omnidirectional radiation. In addition, alumina with a dielectric constant of *ε_d_* = 9.9 and a loss tangent of tanδ = 0.0001 was chosen as the DRA’s material. Figure 1 illustrates the utilized configuration in which the DRA was placed on a square ground plane with a size of *G_s_* = 12.5 mm. The feed network also involved a square Rogers substrate, Ro4003, that was located at the lower side of the ground plane. The substrate had a thickness of *h_s_* = 0.308 mm, dielectric constant of *ε_r_* = 3.5, and loss tangent of 0.0027. Additionally, a 50 Ω microstrip feedline was printed on the substrate’s lower surface with a respective length and width of *l_t_* = 6.25 mm and *w_t_* = 0.3 mm.

The design was evolved by noting that an electrically small vertical probe and a ring-slot that is etched in a metal ground plane represent the duals of a planar loop antenna with equivalent size. Therefore, the small ring-slot provided the same fields as an electrically short vertical probe and, hence, could be considered as an option to create the required planar feeding network that incorporated a rectangular ring-slot as a natural choice to feed a rectangular DRA, as can be observed from Figure 1. However, the ring-slot size may need to be increased depending on the field distribution of the required DRA mode. Furthermore, the utilized ring-slot consisted of *x*- and *y*-directed slot arms with side lengths of *l*_s1_ and *l*_s2_. These slots behave as magnetic currents that excite the required magnetic fields inside the DRA. Since the ring-slot was positioned at the interface between the alumina DRA and the Rogers substrate, the circumference needed to be calculated in terms of the effective wavelength λ_eff_ = λ_0_/√*ε_eff_*, where λ_0_ is the free space wavelength and *ε_eff_* is defined as [24]

(1) $$\varepsilon_{eff}=\frac{{\varepsilon_{d}\varepsilon }_{r}\left(h+h_{s}\right)}{\left(\varepsilon_{d}h+\varepsilon_{r}h_{s}\right)}$$

In order to design an optimum feed, the supported DRA modes need to be identified over the frequency range of interest, as illustrated in the next section.

## 3. Supported Modes of the Proposed DRA

Based on the dielectric waveguide model (DWM) [25], the DRA dimensions were chosen to support the required degenerate modes for omnidirectional radiation at ~28.5 GHz when the DRA is located above a metal ground plane. Therefore, the DRA’s length, width, and height were determined as *a* = *b* = 3.8 mm and *h* = 1.7 mm, respectively. These dimensions offer a compact DRA size that allows easy integration. The resonance frequencies of the ${TE}_{mns}^{y}$ modes can be determined using the DWM as [25]:

(2) $$\begin{array}{c} k_{x}a=m\pi -2\tan^{-1}\left(k_{x}/\left({\varepsilon_{d}k}_{x0}\right)\right)\\ k_{x0}={\left[\left(\varepsilon_{d}-1\right)k_{0}^{2}-k_{x}^{2}\right]}^{\frac{1}{2}} \end{array}$$

(3) $$\begin{array}{c} k_{y}b=n\pi -2\tan^{-1}\left(k_{y}/k_{y0}\right)\\ k_{y0}={\left[\left(\varepsilon_{d}-1\right)k_{0}^{2}-k_{y}^{2}\right]}^{\frac{1}{2}} \end{array}$$

(4) $$\begin{array}{c} {2k}_{z}h=m\pi -2\tan^{-1}\left(k_{z}/\left({\varepsilon_{d}k}_{z0}\right)\right)\\ k_{z0}={\left[\left(\varepsilon_{d}-1\right)k_{0}^{2}-k_{z}^{2}\right]}^{\frac{1}{2}} \end{array}$$

(5) $$k_{x}^{2}+k_{y}^{2}+k_{z}^{2}=\varepsilon_{d}k_{0}^{2}$$

where *k*_0_ is the free space wave number. Owing to the square cross section of the DRA, the resonance frequencies of the ${TE}_{mns}^{x}$ and ${TE}_{nms}^{y}$ modes are equal. Therefore, the required ${TE}_{121}^{x}$ and ${TE}_{211}^{y}$ higher-order modes can be simultaneously excited at the same frequency. This results in a total magnetic field distribution that is similar to that of the HEM_21δ_ mode of a cylindrical DRA that generates an omnidirectional pattern. Table 1 summarizes the supported resonance modes for the chosen rectangular DRA dimensions over a frequency range of 15–30 GHz based on the DWM.

**Figure 2:**
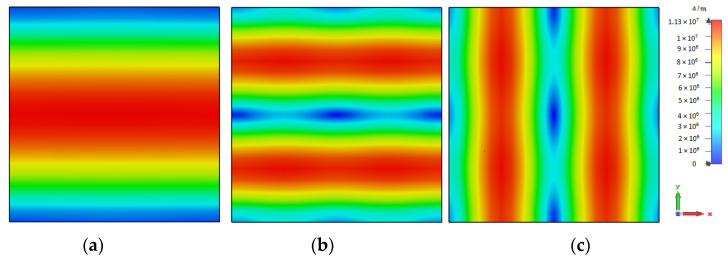
Magnetic field distributions inside the proposed isolated DRA at *z* = 0 when *a* = *b* = 3.8 mm and *h* = 1.7 mm; (**a**)TE_111_ mode at 17.5 GHz, (**b**) ${TE}_{121}^{x}$ mode at 28.5 GHz, and (**c**) ${TE}_{211}^{y}$ mode at 28.5 GHz.

The excitation of the required modes can be achieved by studying the magnetic field distributions of the supported resonance modes inside an isolated DRA which are illustrated in Figure 2. For example, from the ${TE}_{121}^{x}$ mode’s magnetic field distribution, it can be observed that the H-field is null when *y* = 0.5*b*, where *b* is the DRA size. Therefore, the utilization of a centrally located *x*-directed slot aperture will suppress this mode. Hence, the slot needs to be shifted along the *y*-axis to a strong H-field point to excite this mode effectively. Similarly, for the ${TE}_{211}^{y}$ mode, in which the H-field is null at *x* = 0.5*b*, an off-set *y*-directed slot is needed at a strong H-field point for effective mode excitation. However, for omnidirectional radiation, the degenerate modes need to be excited simultaneously. Therefore, a ring-slot aperture was utilized, which involved *y* and *x*-directed slot arms that acted as magnetic current components exciting the aforementioned modes. Furthermore, the chosen DRA dimensions also support the fundamental broadside TE_111_ mode at 17.5 GHz and, hence, it would be beneficial if the same ring-slot aperture excites the fundamental TE_111_ mode as well as the ${TE}_{211}^{y}$ and ${TE}_{121}^{x}$ modes. As mentioned earlier, the interaction between these degenerate modes can result in a field distribution that is similar to the cylindrical HEM_21δ_ mode [22]. For a rectangular DRA, such a mode is defined as a quasi-HEM_21δ_ mode, which offers the required omnidirectional radiation pattern.

Having identified the supported DRA resonance modes and understood the corresponding fields’ distribution, the design of the required ring-slot needed to be implemented as described in the next section together with the achieved DRA performance.

## 4. Design of the Ring-Slot Feed

### 4.1. Square Ring-Slot Feed

**Figure 3:**
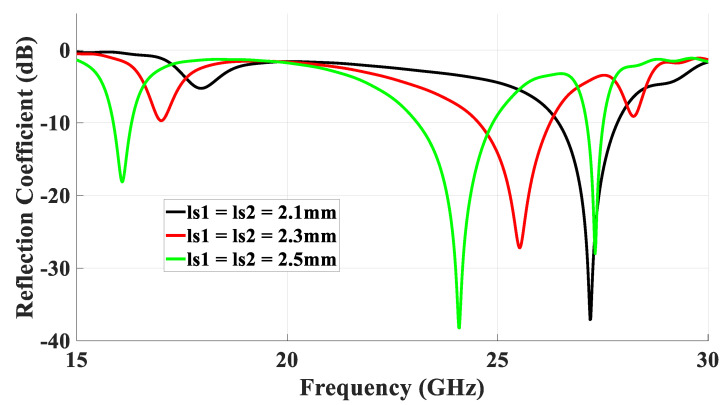
Return losses of the DRA using different sizes of the square-ring feeding slot.

**Figure 4:**
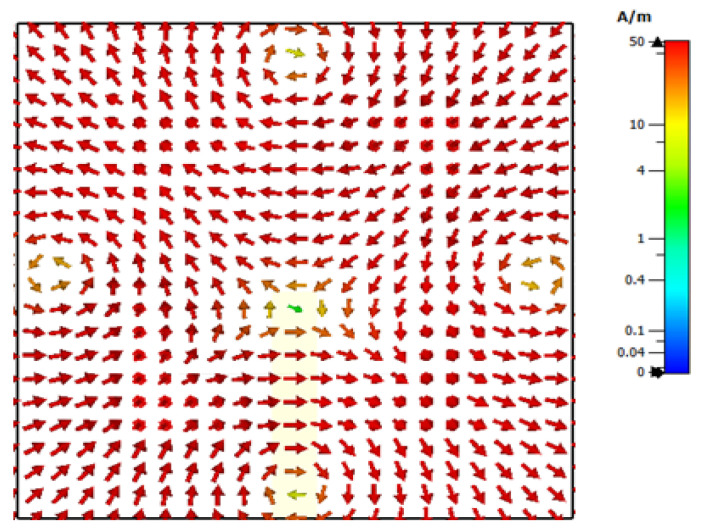
The *xy* plane view of the quasi-HEM_21δ_ internal magnetic field distribution of a square ring-slot-fed DRA at 27.7 GHz.

For simplicity, the special case of a square ring-slot was considered first by setting *l*_s1_ = *l*_s2_. It was important to ensure that the first slot’s resonance, which had broadside radiation, was achieved at a frequency that was different from that of the required ${TE}_{211}^{y}$ and ${TE}_{121}^{x}$ DRA modes to avoid any interference between the different radiation patterns. Subsequently, the separated slot resonance could be suppressed or utilized as another operating frequency band, depending on the design requirements. The return losses are illustrated in Figure 3, where it can be noted that when *l*_s1_ = *l*_s2_ = 2.1 mm and *w*_s_ = 0.5 mm, the slot resonated at 27.7 GHz when the circumference was ~1.1λ_eff,_ which is too close to that of the degenerate modes. This was also combined with a broadside radiation pattern instead of the expected DRA’s omnidirectional pattern, which was not observed initially at the expected frequency. Hence, the size of the slot was adjusted to avoid the coexistence of the DRA and ring-slot resonances at the same frequency. As demonstrated in Figure 3, by increasing the slot size, the DRA modes and slot’s resonance frequencies could be separated as the latter was achieved at 24 GHz when *l*_s1_ = *l*_s2_ = 2.5 mm. Hence, the required omnidirectional and fundamental DRA modes were effectively excited at ~27.7 GHz and 16 GHz, respectively, using the same square ring-slot. In addition, the increased slot size meant the slot arms’ positions could move closer to stronger H-field points of the corresponding DRA’s mode, which resulted in the effective excitation of the required modes. The excited degenerate modes provided an overall impedance bandwidth of 1.3%. Moreover, the TE_111_ broadside mode was excited with a bandwidth of 3%. Moreover, the resonance of the ring-slot was achieved with a bandwidth of 7.4%. It should be noted that the DRA modes were excited at resonance frequencies that were close to those listed in Table 1. Figure 4 presents the simulated magnetic field distribution inside the loaded DRA at 27.7 GHz, which was similar to that of the cylindrical HEM_21δ_ mode, and hence, an omnidirectional radiation pattern was achieved using the rectangular DRA.

**Figure 5:**
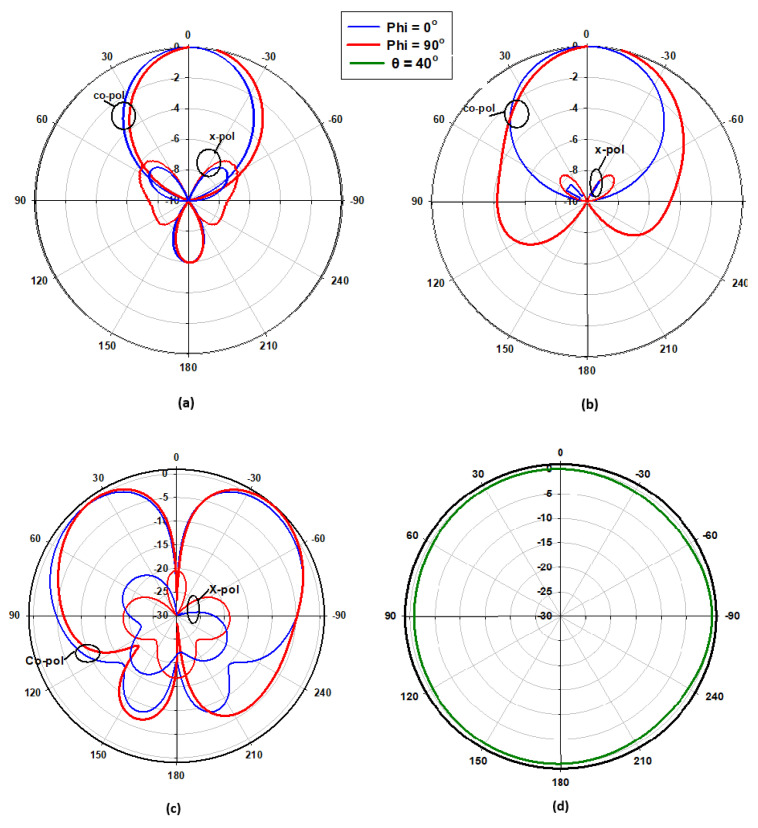
The radiation patterns of square ring-slot fed DRA with arm lengths of *l_s_*_1_ = *l_s_*_2_ = 2.5 mm, (a) 16 GHz, (b) 24 GHz, and (c,d) 27.7 GHz.

**Figure 6:**
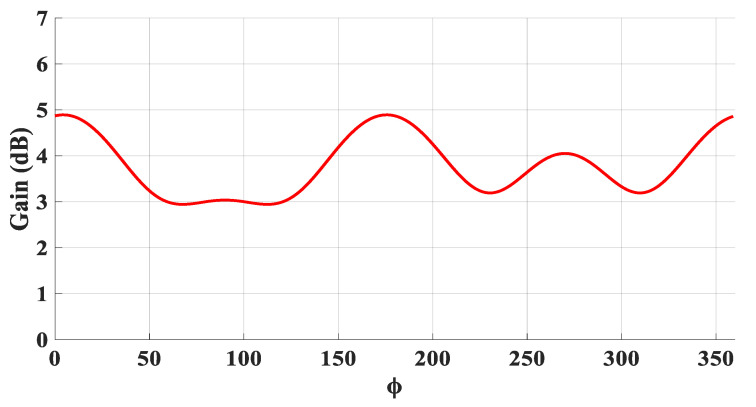
The omnidirectional gain variation at the θ = 40° azimuth plane when a square ring feeding slot is utilized.

**Figure 7:**
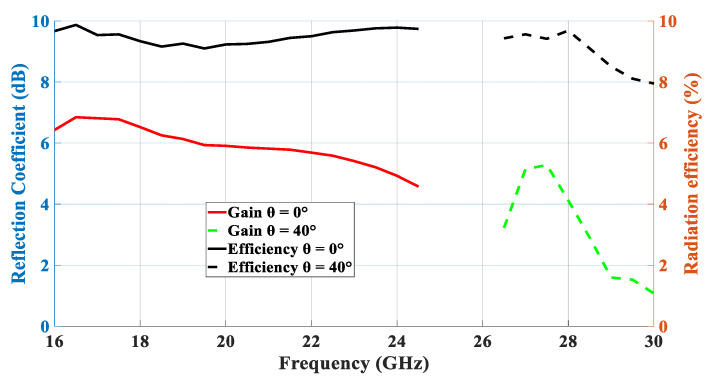
Realized gain and radiation efficiency of the square ring-slot-fed DRA.

Figure 5 presents the achieved radiation patterns at the three resonance frequencies, where broadside radiation patterns were achieved at 16 GHz and 24 GHz due to the excitation of the fundamental DRA mode, TE_111_, and the ring-slot’s resonance, respectively. As mentioned earlier, the quasi-HEM_21δ_ mode was excited at 27.7 GHz when the size of the feeding square ring-slot was increased to 2.5 mm. As demonstrated in Figure 5c, omnidirectional radiation was attained with a maximum gain of 4.1 dBi at θ = 40°. However, a slight asymmetry can be noted in the ϕ *=* 90*°* plane cut, which can be attributed to the asymmetrical feed point position compared to the traditionally used central coaxial probe that naturally enforces the fields’ symmetry. Figure 5d presents the azimuthal patten at the θ = 40° plane, where it can be noted that an omnidirectional pattern was achieved with a modest out-of-roundness. Therefore, a triple-band operation was attained with two different types of radiation patterns using a planar feed network. The variation in the omnidirectional gain at the θ = 40° plane was investigated as illustrated in Figure 6, where it can be noted that a maximum variation of ~1.5 dB exists, which resulted in a pattern that was not perfectly omnidirectional. This can be explained in terms of the limitations imposed by the centrally located square ring-slot, since changing the slot’s size is associated with a proportional shift in the position of each slot arm. This may result in having slot arms positioned at points with slightly different H-field strengths. It should be noted that this variation was slightly higher than that of 1.26 dB for an omnidirectional cylindrical DRA with a planar feed network [20]. An attempt to minimize the omnidirectional gain’s variation is introduced in the next section. Figure 7 illustrates the realized gain at the main beam directions for the three bands. For the two broadside patterns, it can be observed that the realized gains of 6.5 dBi and 4.8 dBi were achieved at 16 GHz and 24 GHz, respectively. On the other hand, an omnidirectional gain of 4.1 dBi was achieved at 27.7 GHz at the main lobe direction of θ = 40°. The simulated radiation efficiency is also illustrated in Figure 7, where it is evident that a high radiation efficiency of ~90% wsa achieved at all the operating frequency bands.

### 4.2. Rectangular Ring-Slot Feed

**Figure 8:**
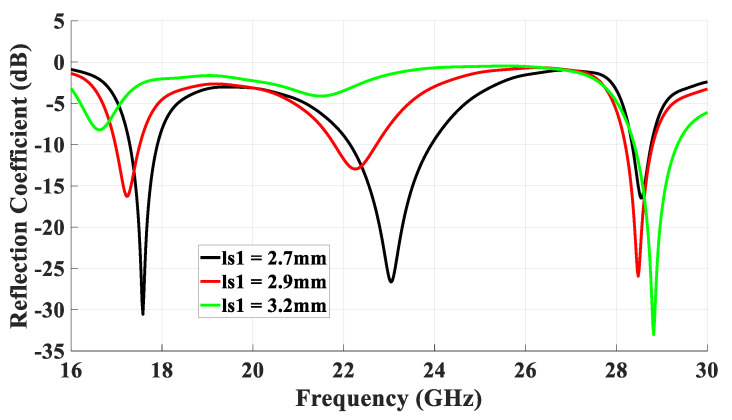
Simulated return losses of the rectangular ring-slot DRA when the longest slot arm’s length, *l_s_*_1_, was varied while *l_s_*_2_ = 2 mm.

**Figure 9:**
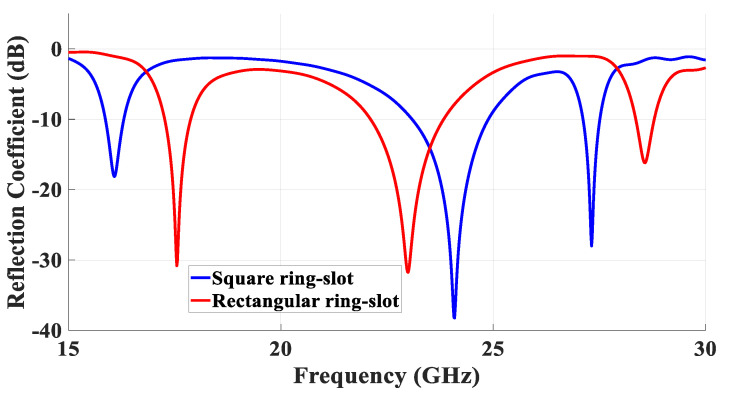
Simulated return losses of the square and rectangular ring-slots fed DRAs.

To minimize the azimuthal gain variations at θ = 40°, a rectangular ring-slot aperture was considered as it offers the flexibility of changing the size of only one pair of the ring-slot arms at a time. The return losses are illustrated in Figure 8 when the longest slot arm’s length, *l_s_*_1_, was varied from 2.7 to 3.2 while *l_s_*_2_ = 2 mm. It should be noted these dimensions resulted in a slot circumference of ~1.1λ_eff_ at 23 GHz. The results demonstrate that the proposed antenna configuration exhibited three operating frequency bands at 17.5 GHz, 23 GHz, and 28.5 GHz for the TE_111_ DRA mode, slot resonance, and quasi-HEM_21δ_ mode, respectively, when *l_s_*_1_ = 2.7 mm. The achieved respective bandwidths for the three resonance modes were 17.3 GHz to 17.9 GHz, 22.1 GHz to 24 GHz, and 28.3 GHz to 28.8 GHz, which correspond to percentage bandwidths of 3.4%, 7.7%, and 1.9%. It can be noted that these bandwidths were wider than those achieved when a square ring-slot was utilized to excite the same DRA modes, which demonstrates the effectiveness of the rectangular ring-slot. It can also be observed from these results that the strongest impact of varying *l_s_*_1_ was on the slot’s resonance frequency, which was expected owing to the change in the circumference of the rectangular ring-slot. On the contrary, smaller variations can be noted in the resonance frequencies of the excited DRA modes as they mainly depend on the DRA dimensions and permittivity. The combination of the reflection coefficient of square and rectangular slots is presented in Figure 9, where it is evident that wider matching bandwidths were achieved when a rectangular ring-slot was utilized.

**Figure 10:**
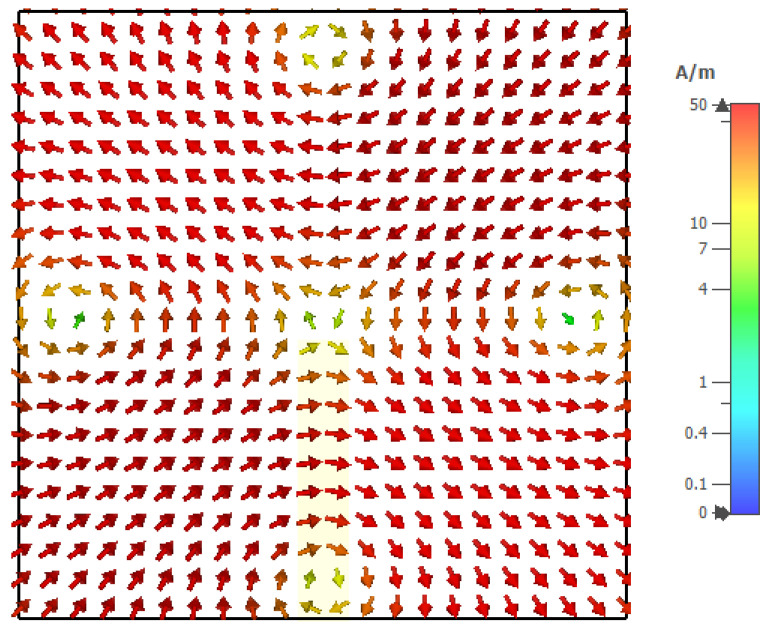
The *xy* plane view of the internal magnetic field distribution inside the rectangular ring-slot-fed DRA, which corresponds to the quasi-HEM21δ at 28.5 GHz.

**Figure 11:**
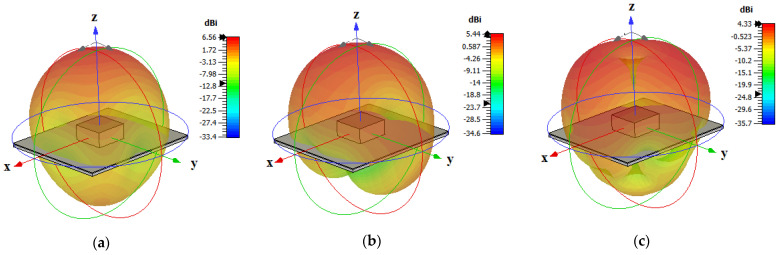
Three-dimensional radiation patterns at the three operating frequency bands: (**a**) 17.5 GHz, (**b**) 23 GHz, and (**c**) 28.5 GHz.

The simultaneous excitation of the degenerate modes was investigated by using a *y*-directed arm slot only with a length of *l_s_*_1_ and an offset of 0.5*l_s_*_2_ from the *x*-axis, which excited the TE_211_ resonance mode at 30 GHz. Similarly, the TE_121_ resonance mode was individually excited at 30 GHz when an *x*-directed slot was utilized with a length of *l_s_*_2_ and an offset of 0.5*l_s_*_1_ from the *y*-axis. However, when the *x-* and *y*-directed linear slots were combined to create the rectangular ring-slot, resonance was achieved at a slightly lower frequency of 28.5 GHz with an overall field distribution that was similar to that of the cylindrical HEM_21δ_ mode, as demonstrated in Figure 10. The 3D radiation patterns for the three operating frequency bands are illustrated in Figure 11, where it can be noted that an omnidirectional pattern was achieved at 28.5 GHz with a maximum gain of 4.3 dBi.

**Figure 12:**
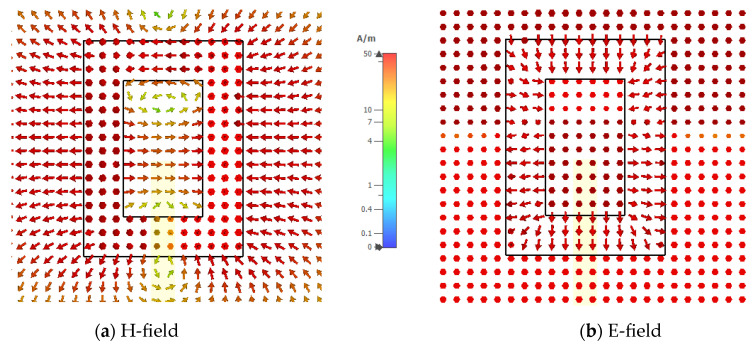
The *xy* plane view of the electric and magnetic field distributions on the rectangular ring-slot feed structure at 17.5 GHz.

**Figure 13:**
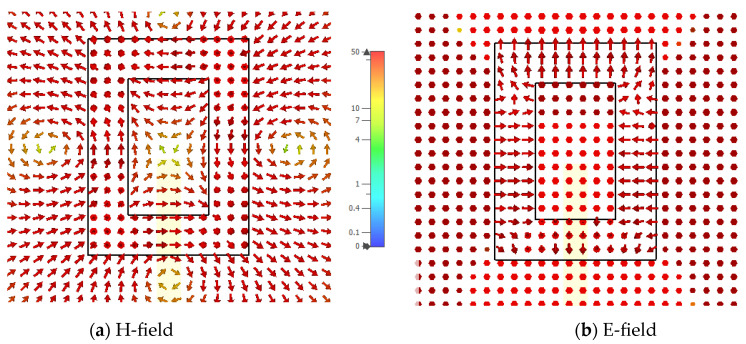
The *xy* plane view of the electric and magnetic field distributions on the rectangular ring-slot feed at 28.5 GHz.

**Figure 14:**
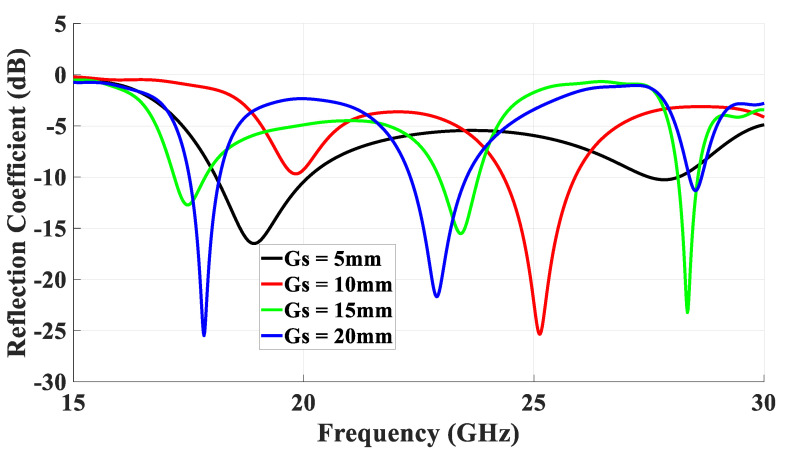
The variation in return losses as a function of the ground plane size.

In addition, the electric and magnetic field distributions on the rectangular ring-slot are illustrated in Figure 12 and Figure 13 at 17.5 GHz and 28.5 GHz, respectively. Figure 14 presents the variation in the return losses as a function of the ground plane size, where it can be observed that the slot’s resonance was strongly dependent on the ground plane size [26]. As a result, there was also an impact on the excited DRA modes and the achieved resonance frequencies due to the variation in the performance of the feeding slot.

**Figure 15:**
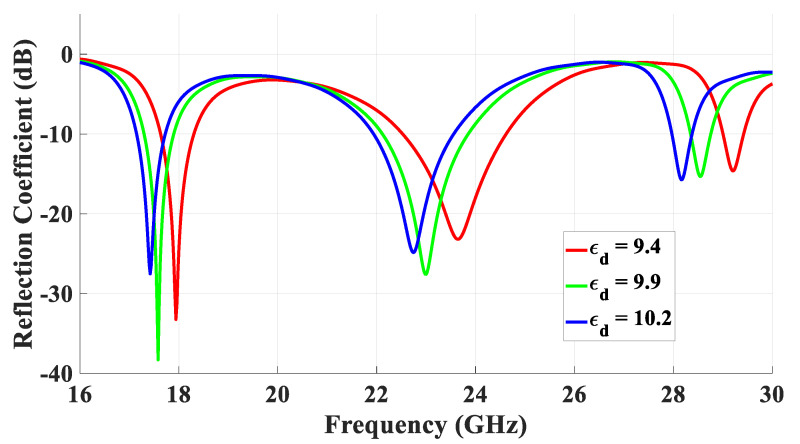
The variation in the return losses as a function of the dielectric constant of the DRA.

Another key parameter that was investigated was the sensitivity of the DRA performance to a range of alumina dielectric constants that have been used in the literature. The results of these investigations are presented in Figure 15, which demonstrates a stable DRA performance when *ε_d_* was varied from 9.4 to 10.2.

The successful design of the omnidirectional DRA is presented in this section. In the next section, the performance of the DRA is investigated in proximity to a human body.

## 5. DRA Performance Next to a Human Body

**Figure 16:**
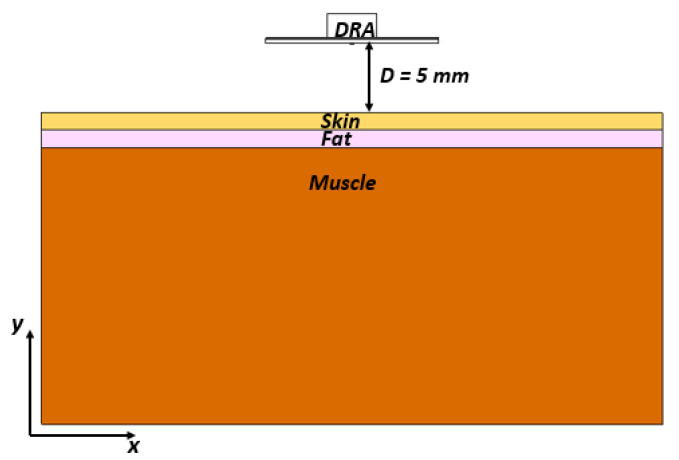
The proposed rectangular DRA in the proximity of a human body phantom.

**Figure 17:**
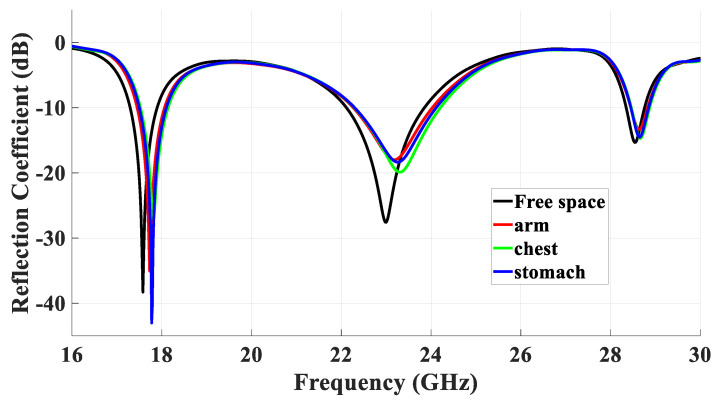
The variations in return losses when the antenna was placed on the arm, chest, and stomach.

**Figure 18:**
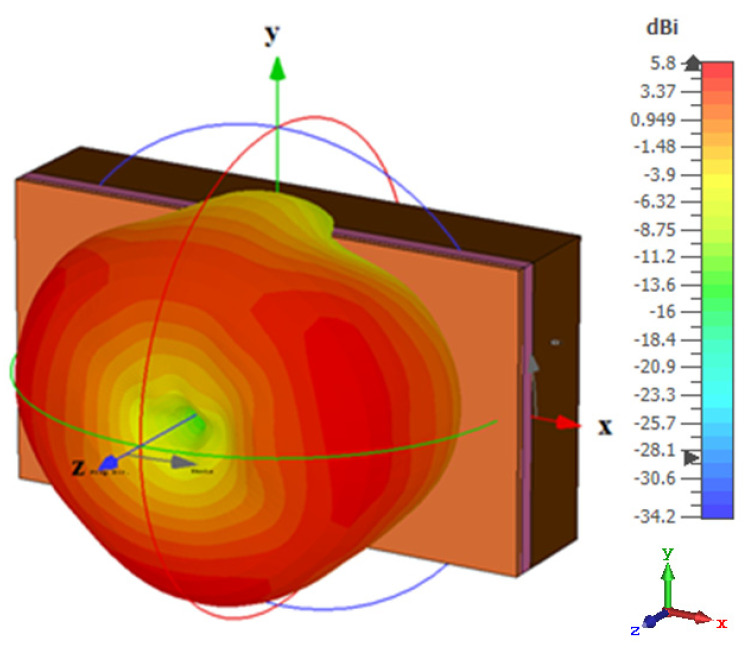
The 3D omnidirectional radiation pattern next to the equivalent tissue at 28.5 GHz when *d* = 5 mm.

For on-body applications, mmWave omnidirectional antennas are widely used. Therefore, it is important that the antenna’s performance is assessed when the proposed antenna is placed next to the human body, as illustrated in Figure 16. In line with the literature, we assessed the DRA’s performance next to three body areas: arm, chest, and stomach [27], where a three-layer phantom was utilized. The utilized parameters for the different tissue layers are illustrated in Table 2. The thicknesses of the three different body parts were based on those reported in [28]. The return losses when the DRA was placed next to arm, chest, and stomach are presented in Figure 17, where it can be noted that the presence of the ground plane minimized the impact of the human body on the resonance frequencies. In addition, the omnidirectional pattern was preserved in the presence of the chest, as demonstrated in Figure 18, which can also be attributed to the presence of the ground plane. However, reflections from the utilized phantom reduced the back lobes considerably and hence increased the omnidirectional realized gain from 4.33 dBi in free space to 5.8 dBi in the proximity of the human body tissue. On the other hand, the presence of the phantom reduced the radiation efficiency from 95% to 84%. However, this did not impact the gain as the increased directivity compensated for any loss due to the slightly reduced radiation efficiency.

**Figure 19:**
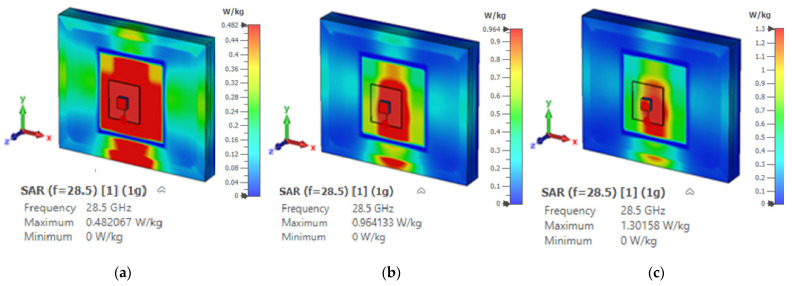
The SAR of the proposed DRA with various input power levels for a 1 g tissue: (**a**) 15 dBm, (**b**) 18 dBm, and (**c**) 20 dBm.

**Figure 20:**
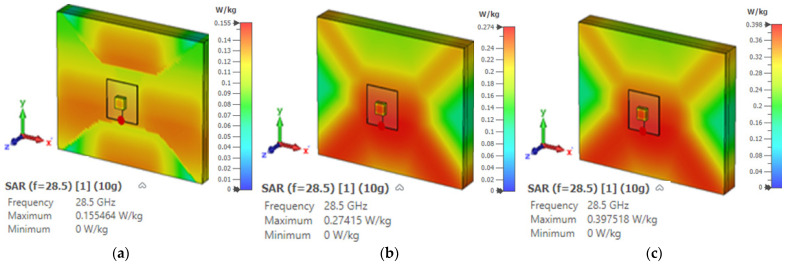
The SAR of the proposed DRA with various input power levels for a 10 g tissue: (**a**) 15 dBm, (**b**) 18 dBm, and (**c**) 20 dBm.

The Specific Absorption Rate (SAR) indicates the safety threshold at which radio-frequency energy can be absorbed by human body tissue [29] The SAR must be assessed to ensure compliance with safety limits set by the Federal Communications Commission (FCC) and the International Commission for Non-Ionizing Radiation Protection (ICNIRP) standards. These standards define SAR thresholds of 1.6 and 2 W/kg for 1 g and 10 g tissues, respectively [29]. Unfortunately, the above guidelines do not offer dosimetric information or suggestions for mmWave frequencies [29,30] However, at 28 GHz, a 5 mm space is recommended between the antenna and the human body with input power levels of 15 dBm, 18 dBm, or 20 dBm at 28 GHz [30]. Subsequently, the proposed omnidirectional DRA was simulated next to a layered phantom, as demonstrated in Figure 16. The conducted SAR simulations confirmed that the radiation from the proposed antenna meets the safety requirements, as illustrated in Figure 19 and Figure 20 for 1 g and 10 g tissues, respectively. It is worth noting that this SRA example is for the scenario of an antenna placed on the chest phantom.

## 6. Measured Results

**Figure 21:**
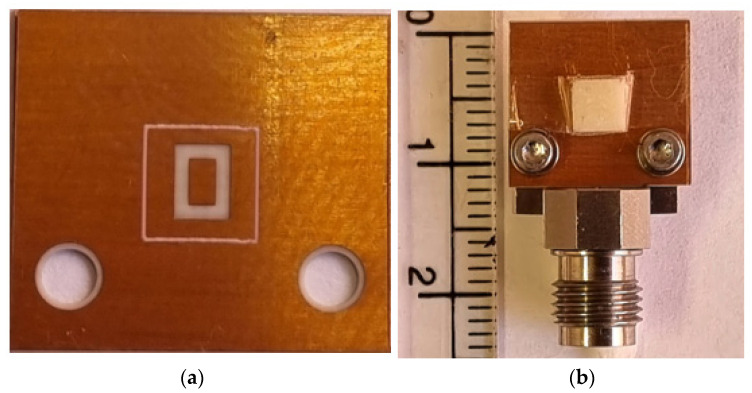
Prototype of the proposed antenna. (**a**) Ground plane with a rectangular ring-slot and outlined DRA position. (**b**) Assembled prototype.

The alumina DRA and planar feed network incorporating a rectangular ring-slot were fabricated by T-ceramics [31] and Wrekin [32], respectively. At the mmWave frequency range, a precise alignment between the DRA and the feeding slot poses significant challenges. To overcome these challenges, a solution involving mapping out the DRA position on the ground plane was implemented during the fabrication stage [33]. The resulting fabricated feed network, which includes the outlined DRA position, is presented in Figure 21a. Following the outlining of the DRA position, ultrathin double-sided adhesive copper tape with a thickness of 0.08 mm was utilized to bond the antenna to the ground plane, ensuring secure assembly. The assembled DRA prototype is presented in Figure 21b, including the utilized ELF50-002 SMA connector that was attached using screws. In addition, the prototype was measured without experiencing any alignment or bonding issues. The implementation of this approach is critical in ensuring the mmWave measurements’ accuracy, where even slight deviations can significantly affect the performance [34]. All measurements were carried out using the UKRI National Millimeter-Wave Facility [35], where an N5245B vector network analyzer (VNA) was employed to measure the return losses following a standard calibration procedure. Based on the analyzed data, the return losses were determined. On the other hand, an NSI-MI Technologies system was utilized in conducting the far-field measurements. By employing this specialized measurement system, various parameters, including the radiation pattern as a function of ϕ and θ, were accurately measured and visualized. To cover the elevation angle range of θ = −90° to θ = 90°, the arm of the NSI-MI spherical system was set up to rotate across the upper hemisphere. Additionally, the gain of the antenna under test was determined with respect to a reference horn antenna.

**Figure 22:**
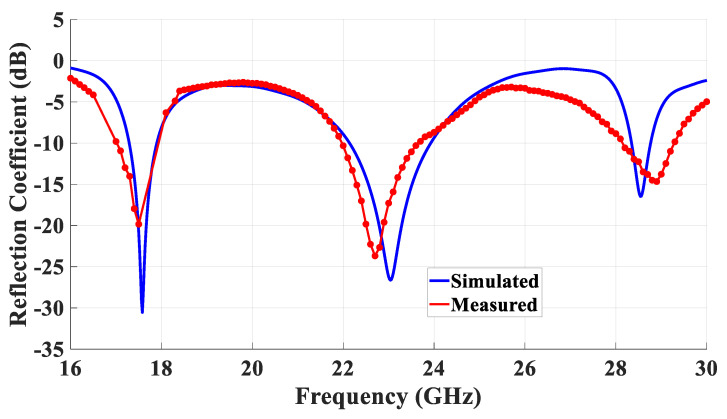
The measured and simulated return losses for the proposed DRA that is fed using a rectangular-ring-slot.

As demonstrated in Figure 22, the measured and simulated return losses shared almost the same resonance frequencies of 17.5 GHz, 23 GHz, and 28.5 GHz for the TE_111_, ring-slot, and quasi-HEM_21δ_ modes, respectively. In addition, the measured and simulated −10 dB impedance matching bandwidth of the lower band was 3.4%. In terms of the middle band that corresponds to the ring-slot resonance, the −10 dB impedance matching bandwidth was 1.8 GHz, demonstrating a good agreement between the measured and simulated percentage impedance bandwidths of 7.7% and 7.5%, respectively. However, a slight discrepancy can be noted in the omnidirectional mode’s simulated and measured bandwidths of 1.9% and 3%, respectively. This discrepancy can be attributed to measurement uncertainties, including measurement setup as well as fabrication and calibration errors. In addition, the utilization of bulky SMA and fittings could have contributed to the discrepancy between simulated and measured results. It should be noted that the achieved impedance bandwidth of the omnidirectional mode was narrower than that of a probe-fed omnidirectional rectangular DRA. For example, impedance bandwidths of 22% were reported in [15] by merging the bandwidths of the DRA mode and that due to the feeding probe’s resonance, which also offers an omnidirectional pattern. However, such a hybrid operation is not possible in the proposed configuration since the feeding ring-slot has broadside radiation, i.e., different from that of the excited DRA mode. Therefore, a feeding ring-slot with an omnidirectional pattern needs to be utilized for bandwidth enhancement. Alternatively, a dielectric coat layer [10], or concentric rectangular ring-slots, can be utilized to achieve a wider bandwidth.

**Figure 23:**
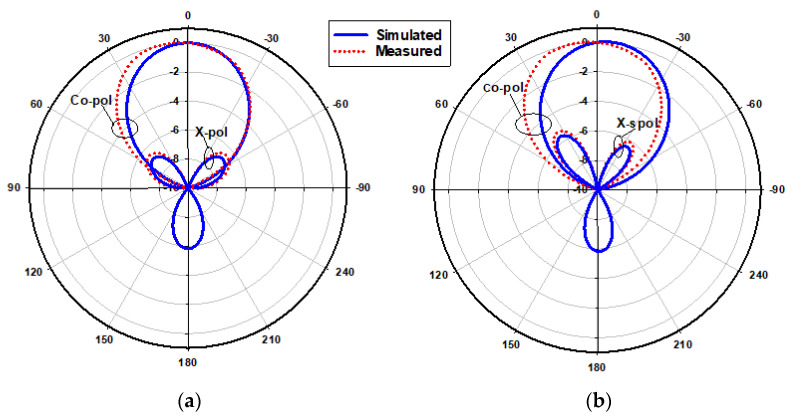
Normalized radiation patterns of the TE_111_ mode at 17.5 GHz: (**a**) E-plane; (**b**) H-plane.

**Figure 24:**
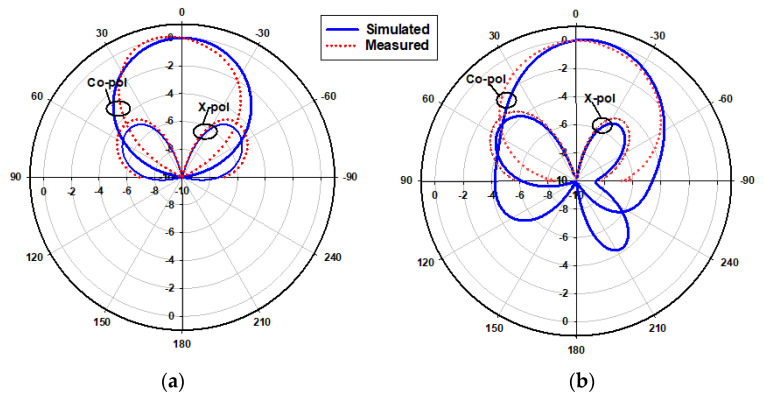
Normalized radiation patterns of the slot mode at 23 GHz: (**a**) E-plane; (**b**) H-plane.

**Figure 25:**
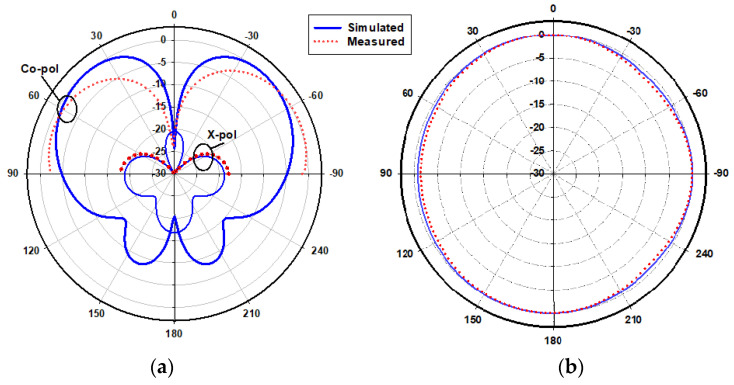
Normalized radiation patterns of the quasi-HEM_21δ_ mode at 28.5 GHz: (**a**) ϕ = 90° plane; (**b**) θ = 40° plane.

**Figure 26:**
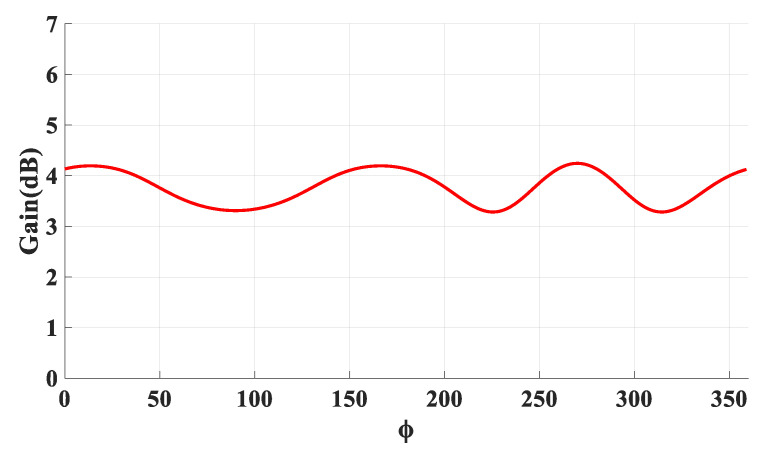
The azimuthal variation in the omnidirectional gain at the θ = 40° plane.

**Figure 27:**
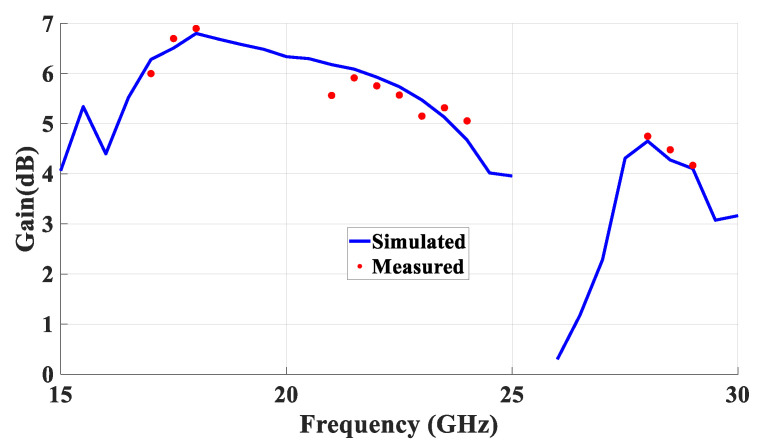
Measured and simulated gains at the main beam directions for the three resonance modes.

Figure 23 presents the measured and simulated radiation patterns at 17.5 GHz, where the TE_111_ broadside mode was excited. Close agreement can be observed between the simulated and measured broadside patterns. As mentioned earlier, the feeding slot’s resonance was achieved at 23 GHz and the corresponding far field patterns are demonstrated in Figure 24, with reasonable agreement between the measurements and simulations. For example, the respective measured beamwidths were 88° and 108° in the E- and H- planes compared to 90° and 107° in the simulations. In addition, the simulated and measured omnidirectional radiation patterns are presented in Figure 25 for both the elevation and azimuth planes at 28.5 GHz. The results demonstrate close agreement between the simulated and measured radiation patterns, where an omnidirectional radiation pattern was achieved with a main beam direction at θ = 40°, as demonstrated in Figure 23a. The measured and simulated beamwidths of the omnidirectional patterns were 61.2° and 60.6°, respectively. However, a slight asymmetry can still be noted in the ϕ = 90° plane cut of Figure 25a, owing to the asymmetrical feed point position. An improved roundness of the azimuthal plane pattern can be observed in Figure 25b, which suggests that the rectangular ring-slot arms were placed at equally strong magnetic field points. Furthermore, the copolarized field component was considerably stronger than the cross-polarized component in all cases. The azimuthal plane gain variation presented in Figure 26, where it is evident that the variation was reduced considerably to ~0.85 dB, resulted in a more stable omnidirectional pattern with close agreement between the measurements and simulations. Additionally, the gain and radiation efficiency of the rectangular ring-slot-fed DRA are illustrated in Figure 27, where it can be noted that the maximum achieved gains were 6.56 dBi, 5.2 dBi, and 4.33 dBi for the TE_111_ mode, ring-slot resonance, and the quasi-HEM_21δ_ mode, respectively. Furthermore, a high radiation efficiency of ~90% was attained in the three operating bands.

A comparative analysis of the ring-slot-fed DRA performance with respect to the reported on-body mmWave antenna designs is presented in Table 3. As mentioned earlier, there is no reported study on the on-body mmWave DRA in the open literature; hence, a comparison was made with respect to different antenna types that are available in the literature. The comparison was conducted with respect to the size, bandwidth, gain, and radiation efficiency. It is evident from Table 3 that the electrical size of the proposed antenna was smaller than most of the reported designs, except that of [36]. In addition, the utilized simple geometry resulted in simple and low-cost fabrication. On the other hand, a triple-band operation was achieved, which was also the case in [36]. owever, the individual bandwidths in the presented design were wider with higher gains compared to those in [36]. At the same time, the other antennas in Table 3 offer single-band operation, albeit with wider bandwidths. Furthermore, the proposed DRA outperformed the reported counterparts in terms of radiation efficiency.

## 7. Conclusions

A multiband millimeter-wave rectangular DRA with different types of radiation patterns was demonstrated. A key achievement was the utilization of a planar feed network to excite an omnidirectional rectangular DRA instead of the traditionally used vertical coaxial probes. The dimensions of the feeding ring-slot were optimized to excite the required resonance modes, with improved performance in terms of the bandwidth and omnidirectional pattern quality. As a result, the quasi-HEM_21δ_ mode was excited for omnidirectional radiation. Moreover, broadside radiation was also achieved by exciting the fundamental TE_111_ mode and the ring-slot resonance mode. It should be noted that all the reported omnidirectional rectangular DRAs operate in the quasi-TM_011_ mode. Therefore, neither a planar feed network nor the excitation of the quasi-HEM_21δ_ mode were demonstrated earlier in the design of omnidirectional rectangular DRAs. Furthermore, an omnidirectional mmWave DRA of any shape has not been reported previously. The omnidirectional mode offers a gain of 4.33 dBi with a notably low azimuthal gain variation of 0.85 dB. The impact of different parts of the human body on the antenna performance was investigated and found to be marginal due to the presence of the ground plane. A comprehensive set of measurements was implemented with close agreement between the simulations and measurements. Overall, the proposed design offers considerable potential for a wide range of applications in the millimeter-wave frequency band. A comparison of the proposed DRA’s performance against those of its earlier-reported counterparts showed that the DRA offers a smaller size and higher radiation efficiency, triple-band operation, and a low-cost, simple design.

## Figures and Tables

**Table 1:** Resonance frequencies of the supported TE modes by the proposed DRA.

Frequency (GHz)	Resonance Mode
17.5	${TE}_{111}$
28.5	${TE}_{121}^{x}$,$\;{TE}_{211}^{y}$

**Table 2:** Human body tissue parameters at 28 GHz [29].

Tissue	Skin	Fat	Muscle
Relative permittivity	16.55	6.09	25.43
Loss tangent	0.2818	0.1454	0.242
Density (kg/m^3^)	1109	911	1090

**Table 3:** Comparison of the proposed on-body antenna’s performance against cutting-edge mmWave counterparts.

Ref	Antenna Type	Frequency GHz	Size (λ^3^) ^1^	S_11_ Bandwidth (%)	On-Body Gain dBi	On-Body η_rad_ (%)
[36]	Slotted patch	28, 38, 61	1.04 × 1.02 × 0.052	3, 1, 1.5	8.1, 8.3, 7	54, 60, 58
[37]	Yagi array	60	3.2 × 1.6 × 0.04	15	9	41
[38]	Patch-like	60	2.8 × 2.1 × 0.23	16.3	12	63
[39]	Textile	28	1.89 × 0.87 × 0.147	33	6.6	53.5
[40]	Q Slot	60	2.58 × 2.8 × 0.32	12	8	56.68
[41]	Patch-like	60	1.6 × 1.02 × 0.23	-	5.4	62.2
This work	RDRA	17.5, 23, 28.5	1.19 × 1.19 × 0.16	3.4, 7.7, 1.9	7.3, 6.8, 5.8	90, 87, 84

^1^ For [36] and this work, λ was calculated at the highest mentioned frequency point.

## Data Availability

Not applicable.
